# 

**Published:** 1899-03-18

**Authors:** 


**Notices of Births, Deaths, and Marriages** are inserted in this column at a charge of 2s. 6d. for an announcement not exceeding four lines.

### NOTICE TO CORRESPONDENTS.

All MS., Letters, Books for Review, and other matters intended for the Editor should be addressed The Editor, "The Hospital," The Hospital Building, 28 & 29, Southampton Street, Strand, W.C.

The Editor cannot undertake to return rejected MS., even when accompanied by stamped directed envelope.

**Notice to Advertisers and Subscribers.**

All Advertisements, Orders for Copies of The Hospital, and Business Communications should be addressed to **THE MANAGER (Not to the Editor),** The Hospital, The Hospital Building, 28 & 29, Southampton Street, Strand, London, W.C.

The Cost of Subscription, post free, is as follows:—Per week, 2½d.; per quarter, 2s. 8d.; per half-year, 5s. 3d.; per annum, 10s. 6d. Foreign:—Per annum, 15s. 2d., payable, in all cases, in advance.

**NOTICE.—Vol. XXIV. of "The Hospital" (April, 1898, to September, 1898), in handsome cloth binding, is now on sale at the Office of this Journal, price 7s. 6d. Cases for binding the Numbers contained in this Volume 1s. 6d. Index to Vol. XXIV., 2½d., post free.**

**The Monthly Part for March is now ready. Price 6d., by post 8d.**

### A NEW COLONY FOR EPILEPTICS.

A new colony for epileptics is about to be established. Mr. Benn W. Levy, acting trustee of the David Lewis Trust, struck by the inadequate provision for epileptics in this country, has been considering the matter for some time, himself visiting the various epileptic institutions on the Continent, and obtaining expert opinions from various sources. The details of the scheme have now been worked out, and land to the extent of 450 acres has been purchased on which to found a "village colony." The total outlay, including the purchase of the site, is estimated at £100,000.

### SCRAPS AND GLEANINGS.

THE total amount received by the Rochdale Infirmary from the Jubilee Endowment Fund has been £24,152 odd.

THE National Telephone Company have granted the free use of their telephone and apparatus to the Rochdale Infirmary, a concession which is much appreciated.

THE appeal by the plaintiffs against the decision of Mr. Justice Stirling in the case of Garton and others v. the Guildford, Godalming, and Woking Joint Isolation Ward in respect to the small-pox hospital now being erected by the board has been dismissed with costs.

IT is intended to enlarge the reception room of the out-patients department at the Birmingham Children's Hospital at an expenditure of £220. The alteration of the whole of the out-patient department is to be commenced shortly, and it is hoped that it may be possible to provide a new operation theatre.

THE report which was adopted at the twenty-sixth annual meeting of the West Cornwall Dispensary and Infirmary contains the following recommendation: That, in consequence of the Workmen's Compensation Act, 1897, in all cases coming within the Act the patients shall be admitted on the employers' ticket, or as private patients at the regular charge of 30s. per week.

IN the course of a few months the extension of the Leith Infirmary is to be begun. About £5,000 has been subscribed through the fund raised in commemoration of the Diamond Jubilee, which is only about one-quarter of the amount required. On completion the hospital will contain 103 beds, besides three isolation rooms, and the nurses' home will provide accommodation for nearly 30 nurses.

LORD BUTE has generously consented to double his gift of £5,000 towards the cost of a new Seamen's Hospital at Cardiff, the only condition attendant on this offer being that the amount of the public subscriptions, which now amount to £7,000, should also be doubled. The Mayor of Cardiff and Colonel Guthrie have each doubled their subscription, thus leaving £4,000 for the public to provide. In addition to this handsome gift Lord Bute is also giving a site for the new building.

### THE STUDENT OF MEDICINE.

### APPOINTMENTS.

H. Blatherwick, L.R.C.P.Lond., M.R.C.S.Eng., appointed Medical Officer for the Dulwich District of the Parish of St. Giles, Camberwell. E. B. Bostock, M.B., B.S.Lond., M.R.C.S., L.R.C.P., appointed Resident Medical and Surgical Officer to the Jaffray Branch of the General Hospital, Birmingham, Gravelly Hill. F. J. Brennand, M.B., C.M.Aberd., appointed Medical Officer for the Fourth District of the Shepton Mallet Union. F. H. Cooke, M.R.C.S., L.R.C.P.Lond., appointed Medical Officer for the Fifth District of the Lexden and Winstree Union. R. N. De Beauvais, L.S.A., appointed Medical Officer for the Milton Abbott District of the Tavistock Union. A. A. G. Dickey, M.D., L.R.C.S.I., appointed Medical Officer for the Colne District of the Burnley Union. J. P. Dunn, M.B , C.M.-Glasg., appointed Medical Officer for the Bishopton District of the Sedgefield Union. F. S. Flint, L.R.C.P., L.R.C.S.Edin., appointed Medical Officer for the Orford District of the Plomesgate Union. R. A. Fryer, M.B., C.M.Edin., appointed Medical Officer for the Hoxton New Town District of the Parish of St. Leonard, Shoreditch. J. Graham, M.B., C.M.Edin., appointed Medical Officer to the Cockermouth Union Workhouse. J. W. F. Graham, L.S.A., appointed Medical Officer for the Brill District of the Thame Union. G. Hillman, L.S.A., appointed Medical Officer of Health to the Castleford Urban District Council. J. J. Hanly, L.R.C.P., L.R.C.S.Edin., appointed Medical Officer for the No. 2 District of the Shepton Mallet Union. F. H. Jacob, M.B.Lond., M.R.C.S., L.R.C.P., appointed House Physician to the Nottingham General Hospital. G. Kempe, M.D., B.S.Durh., M.R.C.S.Eng., L.R.C.P.Lond., appointed Honorary Surgeon to the Salisbury Infirmary. D. G. Kennard, M.R.C.S.Eng., L.R.C.P.Lond., appointed Medical Officer of the Workhouse and the Faringdon District of the Faringdon Union. S. J. J. Kirby, M.D.Brux., L.R.C.P.Edin., M.R.C.S.Eng., appointed Medical Officer of Health to the Hoxne Rural District Council. J. M. Longford, L.R.C.P., L.R.C.S.I., appointed Medical Officer for the Litcham District of the Mitford and Launditch Union. F. W. S. Mann, M.R.C.S., L.R.C.P.Lond., appointed Medical Officer for the Revesby District of the Horncastle Union. C. D. Marshall, F.R.C S., appointed Ophthalmic Surgeon to the Victoria Hospital for Sick Children, Chelsea. J. D. Rawlings, M.B.Lond., appointed Medical Officer for the Northern District of the Dorking Union. G. E. Rennie, M.D., M.R.C.P.Lond., appointed Pathologist to the City of London Hospital for Diseases of the Chest, Victoria Park. G. St. Johnston, M.D.Lond., M.R.C.S., L.R.C.P., appointed Surgeon to the E Division, Birmingham Police.

